# Biosensors in precision livestock farming in dairy production: decoding animals’ needs

**DOI:** 10.5713/ab.260154

**Published:** 2026-03-31

**Authors:** Mingyung Lee, Luis O. Tedeschi, Seongwon Seo

**Affiliations:** 1 Department of Animal Science, Texas A&M University, College Station, TX, USA; 2 Department of Animal Biosystem Sciences, Chungnam National University, Daejeon, Korea

**Keywords:** Biosensor System, Dairy Cattle, Decision Support, Precision Livestock Farming, Reliability

## Abstract

Precision livestock farming in dairy production is advancing through biosensor-based monitoring that converts frequent, longitudinal measurements into actionable information to support animal-level decision-making under commercial conditions. This review summarizes biosensors for precision dairy farming with a systems perspective that connects sensing, data transfer, analytics, and visualization. Biosensors can be categorized by sensing locus as at-animal, near-animal, and from-animal to clarify practical tradeoffs among invasiveness, scalability, maintenance burden, and diagnostic specificity. The review also describes how information should progress from raw signals to interpretable indicators and decision-support outputs, emphasizing that farm value depends on reliable interpretation and timely intervention rather than on measurement alone. Key applications are synthesized across nutrition and feeding behavior, reproduction (including estrus and calving), health monitoring (such as mastitis, lameness, and metabolic disorders), welfare assessment, and environmental sustainability, highlighting where different modalities best support screening, early warning, and confirmatory detection. Finally, the review discusses on-farm barriers, including missing data, sensor drift, attachment stability, communication failures, and alert fatigue, and proposes future directions in standardization, interoperability, and artificial intelligence-enabled decision support to strengthen end-to-end system reliability, scalability, and economic sustainability under commercial conditions.

## INTRODUCTION

Simultaneous demands for economic efficiency, animal welfare, and environmental sustainability are shaping the goals of modern livestock production. These pressures have accelerated the shift in animal agriculture from maximizing production to optimizing production through precision feeding, in which producers are expected to reduce nutrient excretion and environmental impacts while maintaining productivity and welfare-compatible husbandry [1]. This paradigm shift has been extensively reviewed in the context of sensor technology and decision support intelligent tools for smart livestock farming [2], with recent progress highlighting how artificial intelligence (AI)-driven decision support systems have transformed precision livestock farming (PLF) from passive monitoring into predictive management platforms [3]. In this context, PLF has emerged as a practical framework for converting high-resolution longitudinal data into actionable management decisions at the individual-animal level [4,5]. The PLF has been defined as real-time monitoring technologies aimed at managing the smallest manageable production unit, often described as a sensor-based individual animal approach [5]. Reflecting rapid adoption and investment, the global PLF market has been projected to expand markedly over the coming years [6].

Biosensors constitute the technological core of PLF and enable continuous, automated monitoring of animal status and production-relevant phenotypes [7,8]. Early sensor systems in dairy cattle initially focused on reproduction and health monitoring, including estrus detection using activity tags and pedometer-based approaches [9–11]. Over the past decade, biosensors have expanded in both scope and scale, driven by advances in sensing technologies and analytics that increasingly enable the prediction of an animal’s requirements from high-resolution data [5,12]. Accordingly, biosensors in PLF are increasingly recognized not only as classical biochemical devices but also as integrated systems that acquire signals, transmit data, extract information, and present outputs in farmer-friendly formats to support decision-making [7]. Along these lines, the development of biosensors for monitoring dairy cattle is expanding substantially. For example, more than 60 wearable biosensors are commercially available [13]. Taken together, these trends suggest that biosensing is moving toward continuous, automated, and scalable monitoring infrastructures across dairy production systems [8,12].

Despite this progress, synthesizing the evidence base for biosensors in dairy PLF remains challenging because research spans multiple disciplines and reports heterogeneous performance metrics and validation protocols [7,8,12]. Therefore, the objective of this invited review is to provide a systems-level synthesis of biosensors for precision dairy farming by connecting 1) concepts and classifications of biosensors, 2) functional elements and data flow from signal acquisition to information presentation, and 3) phenotype-oriented applications of biosensors in nutrition, reproduction, health, welfare, and environmental sustainability. By integrating technological and animal-science perspectives, this review also aims to clarify current capabilities and limitations and to outline future directions toward reliable, welfare-compatible, and farm-ready biosensing and decision-support infrastructures.

## BIOSENSORS IN PRECISION LIVESTOCK FARMING

### Conventional biosensors and precision livestock farming biosensors

In dairy science, the term biosensor has traditionally been used to refer to analytical devices used for dairy product testing, including milk composition measurements and the detection of residues or microbes. In this classical view, a biosensor is often described as a self-contained integrated device that provides quantitative or semi-quantitative analytical information through a biological recognition element coupled with a transducer [14]. This device-level perspective highlights three core components, namely a biorecognition element (bioreceptor), a transducer, and a power source, followed by signal processing and display [14,15]. The bioreceptor determines selectivity and sensitivity by interacting with the target analyte, while the transducer converts the recognition event into measurable signals that can be processed by an electronic system [14,15].

Biosensors in PLF, however, are primarily designed for data-driven monitoring, enabling continuous measurement and acquisition of physiological and behavioral data from animals under dynamic farm conditions, thereby supporting real-time management decisions. Therefore, the concept of recognition element in PLF needs to be expanded beyond biochemical receptors to include a broader range of sensing modalities and measurable parameters, including video and audio sensors and behavioral patterns [7]. Reflecting this shift, we adopt the following PLF-oriented definition as a key framing statement. Biosensors for monitoring dairy cattle are defined as self-contained or interconnected devices that integrate a recognition element with a signal transducer to detect, measure, and analyze specific characteristics of dairy cattle. This definition encompasses recognition elements, including video and audio sensors, as well as measurable parameters such as behavior patterns.

This expanded definition implies that PLF biosensors should be viewed as components of a monitoring system, rather than as single analytical instruments. First, it accommodates multimodal sensing, in which animal status is inferred from continuous streams of behavioral and physiological signals rather than a single biochemical measurement [7]. Second, it emphasizes the importance of robustness under real-time conditions, which requires careful signal amplification and processing to reduce noise and artifacts and to improve signal-to-noise ratio [16,17]. Third, it draws attention to power and maintenance constraints that are central to on-farm deployment, such as battery limitations in wearable devices, energy-harvesting concepts, and wireless power transfer approaches for implanted sensors [18–20]. Thus, the PLF-oriented definition clarifies why modern biosensing in dairy farming naturally spans biochemical, behavioral, and environmental domains and why it should be framed in relation to decision support rather than solely detection.

### Classification of biosensors

**Figure 1:**
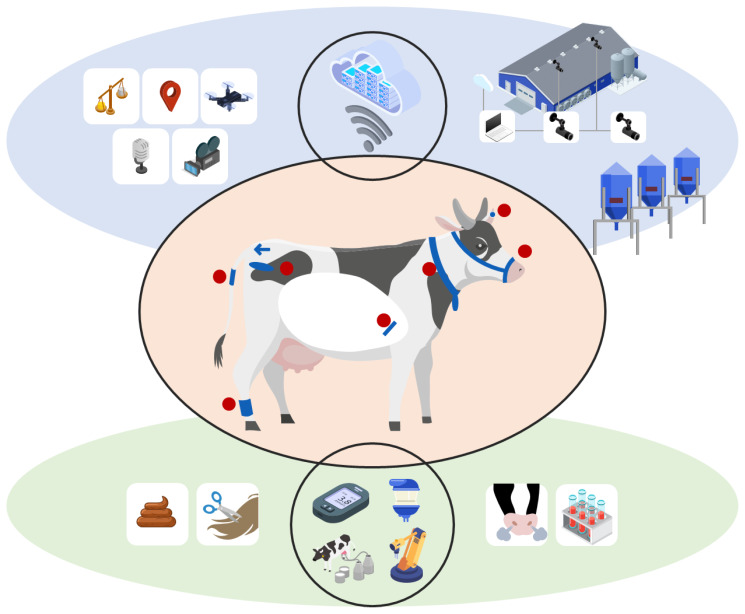
Overview of sensor technologies for monitoring dairy cattle. The orange zone and individual red dots represent ‘at-cattle’ biosensors, the blue zone represents ‘near-cattle’ biosensors, and the green zone represents ‘from-cattle’ biosensors. Adapted from Knight [7] with CC-BY.

The PLF biosensors can be categorized into ‘at-animal’, ‘near-animal’, and ‘from-animal’ sensors, as proposed by Knight [7] (Figure 1). This classification provides a practical framework because it organizes technologies by where signals are acquired and, at the same time, clarifies how each category tends to connect to a distinct management decision point in routine dairy operations. In other words, the value of this classification is not only taxonomic, but also operational, because the type of data produced by each category determines whether the output is best suited for continuous monitoring, large-scale automated screening, or biomarker-based diagnostic confirmation.

#### At-animal biosensors

At-animal biosensors are placed on the exterior or within the animal’s body and are frequently used for continuous tracking of physiological and behavioral parameters. They include wearable, insertable, and implantable devices that are attached to the animal or placed inside the body (orange zone in Figure 1). Wearable systems such as collars, ear tags, and leg bands typically quantify activity and feeding-related behaviors using motion sensors, and some systems incorporate additional physiological sensing capability, such as heart rate. Insertable devices, such as rumen boluses and vaginal sensors, and implantable sensors can capture internal physiological states, such as temperature and rumen conditions, although their adoption may be limited by cost, invasiveness, and maintenance constraints; thus, they might be more adoptable in a research environment. From a management perspective, the decision point of at-animal biosensors is continuous monitoring of behavior and physiology, which enables early warning and timely intervention, for example, estrus alerts, deviations suggestive of health disorders, and patterns consistent with heat stress risk. The practical importance of this category is also reflected in the breadth of commercial availability of wearable biosensors, as summarized in the literature [13].

#### Near-animal biosensors

Near-animal biosensors refer to systems installed in the barn, milking parlor, or farm environment that remotely monitor animal characteristics and environmental conditions (blue zone in Figure 1). These include camera-based systems, acoustic monitoring, and ambient sensors that measure environmental variables such as temperature, humidity, ammonia, methane, and carbon dioxide. Their management decision point is typically non-invasive, scalable automation, which supports herd-level surveillance and routine screening with minimal disturbance to animals, and can also guide environment optimization that affects health, welfare, and productivity. In particular, recent synthesis work highlighted the rapid growth of computer-vision-based monitoring approaches [12]. This trend is consistent with the practical advantages of near-animal approaches, including low animal burden and, in some setups, reduced maintenance when powered via wired connections.

#### From-animal biosensors

From-animal biosensors measure or detect biological or chemical substances in samples such as milk, sweat, blood, breath, saliva, feces, and urine (green zone in Figure 1). In dairy practice, milk is particularly practical because it can be collected frequently during milking, supporting both in-line measurements from continuous milk flow and on-line analyses of collected samples [8]. The management decision point of from-animal biosensors is typically biochemical biomarker-based diagnosis or confirmation, which supports actions such as mastitis detection and treatment selection, ketosis screening, and reproduction management based on endocrine indicators.

Importantly, the three categories should not be interpreted as competing options. Instead, they are often complementary layers in a farm monitoring strategy. At-animal sensors can provide continuous, individualized time-series data that are sensitive to behavioral changes; near-animal sensors can scale monitoring across the herd and environment with minimal burden; and from-animal sensors can add diagnostic specificity through biomarker information.

## ARCHITECTURE OF BIOSENSOR SYSTEMS IN PRECISION LIVESTOCK FARMING

### Functional elements of biosensor systems

**Figure 2:**
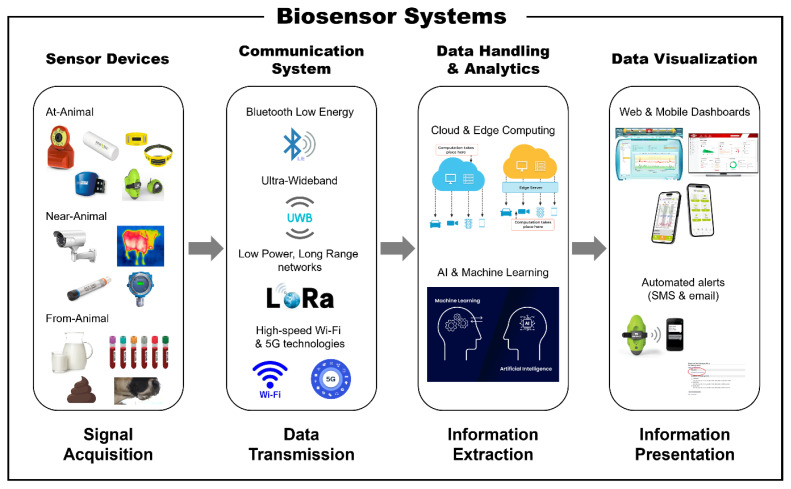
Overview of biosensor systems for dairy cow management and their key components.

Biosensing in PLF should be understood as a system rather than a standalone device, because practical value is created only when signals are transformed into reliable information and delivered in a form that supports management actions. A system-level architecture can be organized into four functional elements: sensing devices, communication systems, data handling and analytics, and data visualization (Figure 2). Operationally, this architecture can be described as a four-step flow in which data move sequentially through signal acquisition, data transmission, information extraction, and information presentation. From this perspective, sensing devices include a receptor, transducer, power source, and internal modules for data processing, reduction, and storage. The data are then transferred to a local or cloud server through a communication system, processed to extract valuable information, and finally summarized and presented in user-friendly formats through visualization.

Within this four-element framework, each layer is associated with distinct implementation requirements and typical failure points on farms. First, sensing converts biological and physical phenomena into digital signals and often requires domain-specific designs, including sample preparation and microfluidics for liquid-sample biosensors [21].

Second, communication determines whether high-frequency monitoring is feasible at scale, particularly in large barns and for devices deployed on or inside the body. Robust transmission can be supported through short-range and long-range technologies such as Bluetooth Low Energy [22], Ultra-Wideband for indoor localization [23], LoRa for low-power long-range transfer, and NB-Internet-of-Things (IoT) for remote biomarker sensors [24]. High-speed Wi-Fi and 5G or 6G connectivity can further facilitate real-time transmission and cloud integration [25], and recent work has highlighted the central role of communication reliability in on-farm deployments [26,27].

Third, analytics transforms raw data into interpretable indicators by combining storage, processing, and model-based inference. Cloud and edge computing support both real-time access and longitudinal analysis, while edge computing can reduce latency and bandwidth by enabling on-site filtering and anomaly detection [28]. Machine learning and AI models contribute to pattern recognition and prediction, including early detection of mastitis and ketosis, estrus monitoring via behavioral changes, and assessment of environmental conditions for climate control and heat-stress mitigation [29,30].

Finally, visualization determines whether outputs are interpretable and trusted by end users. Web and mobile dashboards and on-sensor displays provide real-time insights into health metrics and activity and behavior patterns, while automated reports and notifications can alert farmers via short message service, email, or farm management software [31]. This stack clarifies why PLF biosensing should be evaluated not only by sensing accuracy but also by end-to-end reliability, including data transmission stability, the validity and generalizability of analytics, and the usability of visualization under commercial farm conditions.

### Data-dimension framework

**Figure 3:**
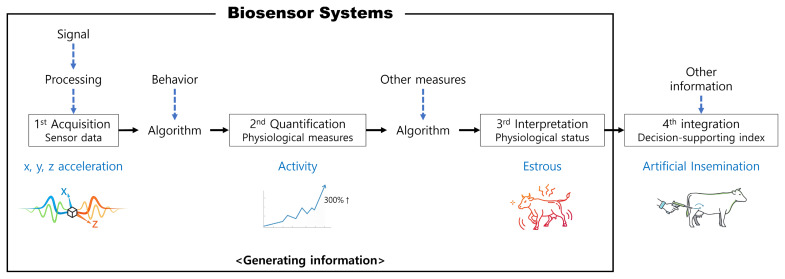
Information-generating process in biosensor systems for dairy cow management.

The system stack describes where data moves and which components enable the pipeline. However, an additional lens is needed to explain how raw outputs become management-relevant decisions. The data-dimension framework provides such a lens by describing an information ladder from raw signals to decision-support indices, including: first, signal and processing; second, acquisition; third, quantification; and fourth, interpretation and integration into a decision-support index (Figure 3).

In this framework, first-dimension data represent raw sensor streams such as x, y, and z acceleration. Second-dimension data quantify derived physical measures, for example, activity indices computed from acceleration. Third-dimension outputs interpret these measures as physiological or behavioral status, such as identifying estrus by integrating activity with other measures, including feed intake and rumination. Fourth-dimension outputs integrate the interpreted information with other datasets, potentially through AI-assisted decision-support systems, to generate decision-support indices such as optimal timing for artificial insemination. The framework also highlights that the core role of PLF biosensor systems is often to generate robust three-dimensional information, because farm decisions depend more on interpreted state than on raw measurements.

This information ladder can be mapped onto the PLF biosensor stack. Sensing and communication primarily generate and deliver first-dimension data. Analytics converts first-dimension signals into second- and third-dimension information through algorithms and model-based inference. Visualization supports fourth-dimension outputs by presenting decision-support indices and enabling actions within farm workflows. We use this framework to structure the review and to compare sensor modalities and phenotypes based on their ability to deliver reliable third- and fourth-dimension outputs.

## BIOSENSOR TECHNOLOGIES AND MEASURED PHENOTYPES

In dairy PLF, biosensor technologies are best organized by the phenotype being measured, because different sensing modalities can capture the same phenotype, and because farm value depends on whether a phenotype supports a clear management decision. Furthermore, many monitoring systems produce continuous data streams, but their practical impact depends on converting raw signals into interpretable animal states and decision-support outputs [31]. Accordingly, this section summarizes biosensor technologies across major phenotype groups and highlights decision-use points, including early warning and anomaly detection. Table 1 summarizes a phenotype-based map of biosensing approaches in dairy PLF, linking typical sensing modalities and sensing loci to decision-use points.

### Behavior and feeding-related phenotypes

Behavioral and feeding-related phenotypes are foundational targets because they directly reflect welfare and productivity and often change before clinical signs become apparent. Common behavioral phenotypes include activity and posture patterns such as activity level, lying time, standing time, and walking behavior, while feeding-related phenotypes include eating, rumination, and drinking behaviors [13]. These phenotypes are frequently captured using at-animal wearable sensors, typically accelerometer-based devices mounted on the leg, ear, or neck, and, in some cases, noseband-based systems that detect jaw movements for quantifying feeding behavior.

Sensor placement and target behavior substantially influence measurement performance. Validation studies and synthesis work have reported high agreement for posture-related behaviors, such as lying and standing, with concordance correlation coefficient (CCC) values exceeding 0.99 for leg-mounted sensors [32,33]. Feeding behaviors also show variable agreement across sensor types and locations; for example, ear tag sensors have demonstrated strong correlations with rumination (r = 0.93) in free-stall systems [32] but variable performance in grazing conditions [34], while neck collars generally show high consistency across systems [35].

From a management perspective, behavioral and feeding phenotypes are valuable for routine surveillance and for identifying deviations from an individual baseline. Deviations in rumination and eating patterns have been associated with metabolic and inflammatory disorders and can therefore support early investigation before overt clinical signs. These phenotypes also connect to precision feeding, where real-time behavioral and production data are used to optimize nutrition management and intervention timing, although fully automated feeding systems remain an area of active development [36].

Near-animal sensing can complement wearables by enabling non-contact monitoring of feeding and behavior through acoustic and camera-based approaches, reducing animal burden and enabling scale to herd-level surveillance. The decision-use points in this phenotype group include ration adjustment, early follow-up on abnormal feeding patterns, and prioritization of animals for health checks.

### Health-related phenotypes

Health phenotypes in dairy PLF often emphasize early warning and anomaly detection, because many economically important disorders are best managed when deviations are detected early and interventions are applied promptly.

Lameness is a major target for both welfare and productivity reasons. Wearable approaches can capture changes in activity patterns and locomotion-related proxies, while vision-based approaches quantify gait and posture features such as stride length, step symmetry, walking speed, and back curvature [37–39]. Thermal imaging has also been explored for health-related phenotypes, including lameness-associated indicators [40]. These systems support decision points such as screening for suspected lameness, prioritizing animals for hoof inspection, and triggering timely veterinary intervention.

Mastitis can be readily monitored using from-animal biosensors because milk is routinely available. Biomarker-based approaches have targeted acute-phase proteins and enzyme activities, including haptoglobin and NAGase, with immunosensor and assay developments reported in the literature [41–44]. Pathogen detection approaches include DNA-based platforms and portable qPCR-based systems for major mastitis pathogens, supporting rapid decision-making for treatment selection and infection control [45–47]. In automatic milking systems, real-time monitoring often incorporates electrical conductivity and somatic cell count as practical indicators for mastitis screening and alerting [48].

Ketosis and metabolic disorders are also well suited to from-animal sensing. Milk β-hydroxybutyrate monitoring has been reported as a practical approach for diagnosing and screening for ketosis [49]. Near-animal systems can contribute through automated body condition scoring, which supports metabolic risk management by detecting abnormal changes in body reserve [50,51].

Heat stress can be monitored by combining animal-level temperature sensing and ambient sensing. Internal temperature monitoring using insertable devices has been used to track physiological responses relevant to heat stress, and near-animal approaches, such as thermal imaging, have been investigated for heat-stress phenotypes [52].

### Reproductive phenotypes

Reproductive phenotypes remain central to PLF because estrus detection and calving prediction directly affect reproductive performance and labor allocation. Camera-based systems are increasingly used for estrus-related behaviors, including mounting detection using deep learning approaches, with reports of high performance for continuous monitoring under farm conditions [53–56]. Such systems can reduce missed heats and support decision timing for insemination, especially when integrated with complementary phenotypes.

At-animal sensing also plays a major role. Vaginal temperature monitoring has been reported to detect estrus, including cases with weak behavioral expression, and has been used for calving prediction through characteristic temperature changes and expulsion-based alerting approaches [57–60]. From-animal sensing provides endocrine information, and milk progesterone monitoring has been developed for reproductive decision support, including in-line progesterone biosensor concepts [61]. The key decision-use points in reproduction phenotypes include estrus alerting, optimizing insemination timing, and prioritizing calving supervision.

### Environmental and sustainability phenotypes

Environmental phenotypes are increasingly important because they connect animal welfare and productivity to sustainability outcomes. Near-animal ambient sensors can quantify barn temperature and humidity and can support indices such as the temperature-humidity index (THI). Ambient gas sensors can also measure air quality-related variables such as carbon dioxide, ammonia, and methane, supporting ventilation control and emission monitoring. These measurements directly inform management decisions, such as heat abatement strategies, ventilation optimization, and assessments of emission mitigation practices, and become more informative when interpreted in conjunction with animal-level behavior and physiological indicators.

## REPRESENTATIVE SENSOR SYSTEMS BY CATEGORY

This section summarizes representative biosensor systems by category, emphasizing their advantages, constraints, and recommended farm settings. The goal is to provide an application-oriented synthesis that helps match sensor systems to management objectives and farm infrastructure.

### At-animal biosensor systems

Wearable systems (e.g., ear tag, neck collar, leg band, noseband, and chest band) are widely adopted because they support continuous, individual-level monitoring of behavior and physiology, and more than 60 commercially available products were reported [13]. Their key advantage is the ability to generate dense time-series data for activity, rumination, and feeding behavior, which are central to estrus detection and early warning of health or nutritional disorders.

Ear tag sensors combine identification with head and jaw movement monitoring and can incorporate temperature sensing, enabling simultaneous tracking of behavior and surface temperature signals relevant to stress, health, or estrus [34,62,63]. A practical constraint is retention, because ear tags may fall out and require replacement, which increases labor and missingness in longitudinal datasets. Ear tags are often recommended when farms want a lightweight device that can add temperature information in addition to activity and feeding proxies.

Neck collar sensors provide stable mounting and are widely commercialized. They are useful for monitoring eating and rumination via repetitive head movements and for activity-based inference related to estrus and health deviations [35,64]. Their main constraints are sensitivity and specificity for certain behaviors because head movement may not always map cleanly to specific activities, and stable placement can be an issue in some farms. Neck collars are often a good choice in free-stall systems when the primary targets are rumination and general activity trends, and when on-animal maintenance needs to be minimized.

Leg band sensors are commonly used as pedometer-type devices and are particularly useful for locomotion-related phenotypes and lameness screening because they capture gait and mobility proxies with high resolution [65,66]. A common constraint is environmental contamination, as leg tags can become covered in mud or manure, which can affect performance and require cleaning or maintenance. They are recommended when locomotion monitoring is a priority and when barn hygiene and tag cleaning logistics are manageable.

Noseband sensors directly quantify jaw movements and can achieve high precision for chewing and rumination detection, making them strong tools for feeding behavior monitoring and early detection of deviations linked to digestive and metabolic health [64]. Their constraint is that they require correct fitting and may be less convenient than collars or ear tags in some farm workflows. They are recommended when feeding behavior, rumination, and early nutrition-related alerts are core objectives, particularly in research herds or farms with a strong interest in precision feeding.

Chest band sensors offer a less invasive alternative to full electrocardiography systems for heart rate and heart rate variability monitoring, which can support stress and welfare-related applications [67]. Their constraints include signal artifacts due to motion and the need for reliable skin contact. They are recommended for targeted monitoring periods, validation studies, and welfare research where cardiovascular measures are needed but surgical implants are not appropriate.

Insertable systems (e.g., rumen bolus, vaginal sensor, and nasal ring) provide access to internal or semi-internal signals and can be more robust to external noise, but they introduce constraints related to cost, calibration drift, comfort, and data transmission through tissue and body blocking.

Rumen bolus sensors are stable in the rumen or reticulum and allow continuous internal measurement of rumen pH and temperature, supporting early detection of subacute ruminal acidosis and monitoring of heat stress or febrile illness [68–70]. Their constraints include higher cost, pH sensor replacement needs due to calibration drift, and limitations for broad adoption outside research or high-value herds [69,71]. They are recommended when rumen physiology is a key target, such as transition cow monitoring, nutritional intervention trials, or high-risk herds needing early detection of digestive disorders.

Vaginal sensors are primarily used for reproductive monitoring, including estrus detection and calving prediction via continuous vaginal temperature measurement and expulsion-based alarms [57,59,60]. Constraints include communication dropouts due to body blocking, individual variability that may cause false alerts, and potential mild discomfort in some cows [72,73]. They are recommended when calving supervision is a major management bottleneck, such as in farms with limited night labor, dystocia risk, or large herds where targeted calving alerts improve welfare and labor efficiency.

Nasal ring sensors represent a minimally invasive approach for monitoring feeding and rumination behaviors and can leverage machine learning models for classification [74]. Emerging designs have incorporated photoplethysmography to estimate heart rate and respiratory rate in free-moving cows, showing strong agreement with electrocardiography references and high respiratory-rate detection performance [75]. Constraints include the need for secure attachment and robustness of wireless transmission. These systems are recommended as emerging options when farms or research programs aim to capture jaw-movement signatures without full noseband setups.

Implantable sensors can provide continuous monitoring of core physiological parameters such as core temperature, glucose, and cardiovascular signals, but they are typically constrained by invasiveness and cost and are therefore more common in research or specialized high-value contexts. For example, subdermal temperature sensors implanted near the base of the ear have shown close agreement with vaginal temperature while enabling frequent wireless transmission [76], and fully implantable wireless thermometers embedded in muscle have remained stable over at least one month [77]. Continuous glucose monitors originally developed for humans have also been explored for tracking glucose trends in cows, although absolute accuracy can be limited [78]; related work in sheep suggests that multi-day subcutaneous glucose tracking is feasible in ruminants [79].

### Near-animal biosensor systems

Near-animal systems are attractive because they can reduce animal burden and can scale to herd-level surveillance, particularly when installed infrastructure can be powered via wired connections. This category includes automated weighing, camera-based computer vision, acoustic monitoring, and ambient environmental sensing, each supporting distinct decision points.

Walk-over-weight systems and scales enable routine body weight monitoring and can achieve very high agreement with manual measurements after appropriate data processing [80]. Their advantages include low animal disturbance and straightforward interpretation of weight trends for health and production management. Constraints include installation requirements, traffic flow design, and data cleaning for repeated passes. These systems are recommended in farms with consistent cow flow patterns, such as near milking parlor exits or robot milking traffic lanes.

**Figure 4:**
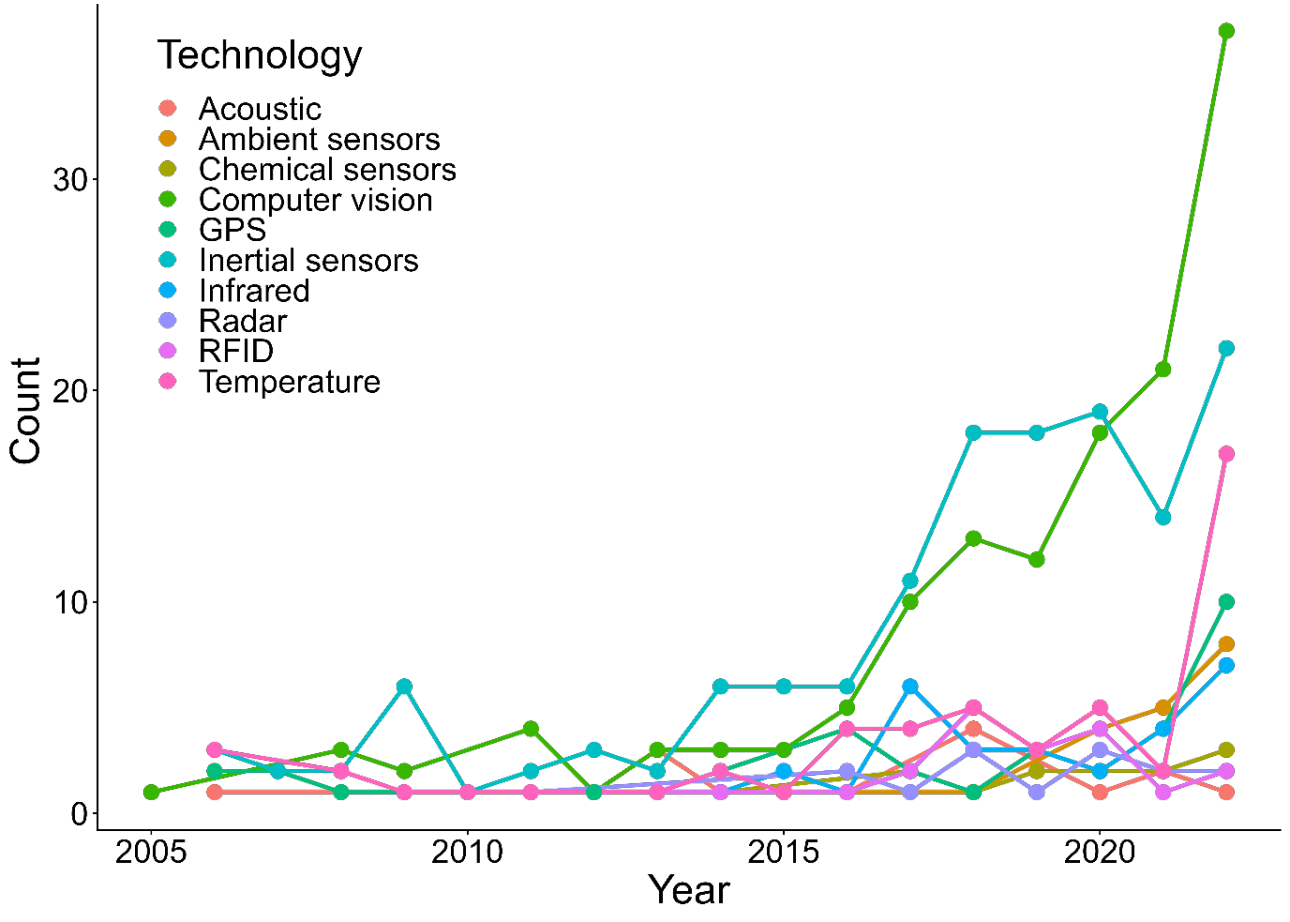
Trends in biosensor technologies for dairy cattle monitoring. Adapted from Besler et al [12] with CC-BY.

Camera-based systems (2D, 3D, closed-circuit television, thermal, light detection and ranging technology) support a wide range of phenotypes and are a major growth area in cattle monitoring. A re-analysis of Besler et al [12] indicates that computer vision increased from 21.4% of biosensor technologies in 2015 to 31.6% in 2022, reflecting rapid expansion and positioning machine vision as a dominant technology (Figure 4). Recent reviews have synthesized specific deep-learning algorithms applied to cattle computer vision, including convolutional neural networks, YOLO-based object detection, and image segmentation for predicting individual feed intake, body weight, body condition score, health status, and reproductive performance [81]. The broader integration of these AI-driven computer vision tools into multimodal sensor fusion frameworks, digital twin technologies, and edge computing architectures has been examined in the context of challenges related to data quality, model generalizability, and the need for explainable, human-centered design approaches to support adoption [82]. Advantages include non-invasive monitoring, automated dataset generation, and reduced battery constraints when powered through wired connections, which can lower maintenance and labor relative to many on-animal devices. Thermal imaging has been applied to phenotypes including heat stress, estrus-related monitoring, and lameness-related indicators [40,52,56]. The light detection and ranging technology has been used for body weight and biometric measurements [83,84], while 2D and 3D cameras have been applied to body condition scoring, lameness detection, and estrus monitoring [37,55,85]. For estrus detection, a background subtraction approach in tie-stall cows reported 90% sensitivity [54]. Constraints include installation cost, line-of-sight issues, lighting variation, bandwidth and storage demands at high sampling rates (especially for multi-camera deployments), and the need for robust algorithms that generalize across barns and seasons. To maintain communication reliability at scale, many implementations rely on on-site (edge computing) processing and selective transmission of features or event-level alerts rather than continuous raw-video streaming. Camera-based systems are recommended in free-stall barns and areas with predictable cow movement, such as alleys, feeding lines, milking parlor entry lanes, and robot milking stations, where consistent viewpoints support stable analytics.

Acoustic systems can quantify mastication and rumination sounds and can also use vocalization signatures as proxies for stress, discomfort, or disease. A notable example is cough-sound based respiratory disease detection in calves using the SoundTalks platform, where microphones installed in calf pens continuously recorded cough sounds and an algorithm was evaluated against veterinarians’ clinical respiratory scores [86]. In that study, the system achieved 50.3% sensitivity and 99.2% specificity, indicating low false-positive burden with moderate case capture, and suggesting usefulness as a rule-in tool for pen-level surveillance and early warning. Constraints include barn noise, microphone placement, and label quality for training models. Acoustic systems are recommended for barns where stress and respiratory health monitoring is a priority and where sensors can be installed in stable locations.

Ambient sensors measure temperature, humidity, and gases such as carbon dioxide, ammonia, and methane, supporting outputs such as THI and air quality monitoring. Advantages include continuous environment surveillance and direct links to ventilation and heat abatement decisions. For example, Flessner et al [87] evaluated a temperature/humidity data logger under cattle-barn field conditions and reported an area under the receiver operating characteristic curve of 0.99 to 1.00 for classifying heat-stress conditions using a THI cut-off of 72, indicating very high agreement with comparator devices. Constraints include sensor drift, spatial heterogeneity within barns, and the need to interpret values in relation to animal-level responses. These sensors are recommended as baseline infrastructure in most modern barns, particularly where heat stress and air quality are persistent risks.

### From-animal biosensor systems

From-animal biosensors measure biological or chemical substances in biological samples, and in dairy systems milk is particularly practical because it can be collected at least twice daily during milking, enabling routine monitoring. The main advantage of this category is higher biological specificity, which supports diagnostic and confirmatory decisions, particularly for metabolic status, reproduction, and udder health.

Milk-based sensing includes biomarkers for ketosis and energy balance, where milk β-hydroxybutyrate supports diagnosis and screening [49]. Reproductive monitoring can use milk progesterone for estrus and pregnancy detection, and in-line progesterone biosensing has been developed [61]. Mastitis detection can use milk biomarkers such as haptoglobin and NAGase and can also include DNA-based pathogen detection approaches [41,42,45]. In automatic milking systems, real-time monitoring often incorporates electrical conductivity and somatic cell count for mastitis screening and alerting [48]. Constraints of milk-based biosensing include the need for standardized sampling and calibration, potential interference from milk composition variability, and integration costs with milking infrastructure. Milk-based sensing is recommended when farms have milking infrastructure that supports routine sampling, particularly in robot milking systems and high-throughput parlors where automated sampling is feasible.

Other biological samples such as sweat, saliva, feces, and urine are being explored as non-invasive matrices for biosensing, with ongoing development for practical farm deployment [88]. Their advantages include expanded biomarker opportunities and potential for non-milking animals or non-lactating groups, while constraints include sampling logistics, lower routine frequency than milk sampling, and the need for robust on-farm assays. These approaches are recommended primarily in research, targeted diagnostic contexts, or future integrated platforms where sampling can be automated or coupled with existing workflows.

## PERFORMANCE EVALUATION FOR ON-FARM RELIABILITY

Performance evaluation in PLF biosensing should focus on reliability in practice, meaning whether a system maintains consistent decision quality under real farm constraints such as variable environments, missing data, and long-term deployment. Practical reliability depends not only on the sensing element, but also on stable placement, resistance to harsh conditions, long-term consistency, and the availability of reference data for validation. This perspective is especially important because false alarms and missed events carry asymmetric costs, and decision-support systems are ultimately judged by whether they reduce labor and improve outcomes without creating alert fatigue or workflow disruption.

### Key evaluation metrics

For PLF biosensor systems that generate alerts for events such as estrus, mastitis, ketosis, lameness, or respiratory disease, performance evaluation should begin with sensitivity and specificity, but it should not end there. Sensitivity quantifies the probability that a true positive animal is detected, and specificity quantifies the probability that a true negative animal is not incorrectly flagged. While these two metrics are fundamental for method comparison, farmers and field veterinarians experience system performance primarily through the quality and burden of alerts, which is captured more directly by positive predictive value (PPV) and negative predictive value (NPV).

A critical point is that PPV and NPV are not intrinsic properties of the sensor alone. They are functions of prevalence, meaning the underlying frequency of the event in the target population and time window. When prevalence is low, even a system with high sensitivity and high specificity can generate a large number of false positives relative to true positives, causing PPV to drop and increasing the operational burden of checking alerts. This is why reporting only sensitivity and specificity can be misleading for adoption decisions. It can also explain the common field observation that systems perceived as accurate in trials can still create alert fatigue in commercial farms.

The worked example presented for 100 animals illustrates this logic. With prevalence 20%, sensitivity 90%, and specificity 95%, the PPV is 82%. This implies that approximately one in five alerts (4 of 22) will still be false alarms, and some true cases (i.e., two animals) will still be missed (false negatives). If prevalence decreases, PPV declines further even when sensitivity and specificity are unchanged. Therefore, when a phenotype has low prevalence in a given period, specificity often becomes the dominant determinant of practical usability, because a small drop in specificity can create a large absolute number of false positives in a population where most animals are negative.

This prevalence effect is also closely related to threshold selection in alerting models. In practice, moving the threshold to increase sensitivity usually decreases specificity, which may be acceptable for time critical events where the cost of a missed event is high, but it can be unacceptable for low-prevalence conditions where false alerts rapidly accumulate. For this reason, studies should report not only point estimates of sensitivity and specificity but also threshold-dependent summaries, such as the expected number of alerts per day or false positives per 100 cow-days, at a clearly stated operating threshold. These operational metrics translate statistical performance into workflow impact, which is central to reliability in practice.

For continuous or time budget phenotypes such as rumination time, eating time, lying time, or weight trajectories, correlation alone is insufficient. Agreement and error metrics, including CCC and root mean square error (RMSE) or RMSE of prediction (RMSEP), provide more informative evaluation because they quantify both precision and bias, which determine whether outputs are usable for decision thresholds rather than only for descriptive monitoring. In addition, the evaluation should explicitly define the intended use case. For example, if the goal is early warning based on deviation from baseline, the most relevant question is whether the system can detect meaningful change reliably, not whether it perfectly matches absolute reference values. High agreement metrics in real deployments can be achieved for some quantitative traits with appropriate processing. For instance, walkover weighing has reported CCC close to 0.99 after data processing, illustrating the value of agreement-based reporting for practical feasibility assessment.

Therefore, reliability-focused performance reporting in PLF should require event-based systems to report sensitivity, specificity, prevalence, PPV, and NPV, accompanied by expected alert burden at the chosen threshold. For continuous prediction, CCC and RMSE (or RMSEP) should be reported with bias considerations, and the interpretation should be tied to the intended management decision, such as screening, confirmation, or intervention triggering. These recommended metrics and operational reporting items are summarized in Table 2.

### Determinants of performance under farm conditions

Reliability in practice is shaped by a set of recurring factors that are often underreported but strongly determine whether an algorithm that performs well in trials remains useful on farms.

Housing and production context can change the mapping between sensor signals and the target phenotype. For example, activity and rumination correlations can differ across tie-stall, free-stall, and grazing contexts, reflecting differences in mobility, terrain, and daily routines, and this can create substantial performance variability if models are transferred across systems without adaptation [33,34]. Visual monitoring can also be constrained by the environment. Occlusion in crowded pens and restricted movement can reduce the expression or visibility of behaviors such as mounting, making camera placement and barn layout critical determinants of estrus detection performance [54,55].

Sensor placement and attachment stability often dominate long-term reliability for at-animal systems. Wearable devices can be covered by mud or manure and may detach or fall out, producing systematic missingness that degrades both monitoring and model calibration over time. Stable placement is repeatedly emphasized as a requirement for improving reliability and long-term consistency. Contamination and maintenance needs are especially important for near-animal systems. For automated weighing platforms, frequent cleaning of manure from the platform is necessary to ensure accurate measurement, and installation at multiple stations can be costly. Even with accurate measurement hardware, reliable operation depends on traffic flow design, outlier detection, and how daily representative values are defined from repeated passes.

Communication interruptions and data loss can be a major driver of real-world underperformance, particularly for internal sensors and systems operating in large barns. Communication dropouts due to body blocking have been reported for vaginal sensors, underscoring that transmission reliability is part of biological sensing reliability, not a separate engineering detail [73].

Sensor drift and calibration requirements can limit long-term deployment for biochemical or internal sensors. For example, rumen bolus systems can operate for years for some channels, but pH sensors may require replacement due to calibration drift [69]. Drift is therefore a core reliability issue and should be quantified and reported, because it directly affects whether models trained early in deployment remain valid months later.

Gold standard definition and reference data acquisition remain fundamental bottlenecks. Even for seemingly straightforward phenotypes like body weight, accurate reference measurement can be challenging due to diurnal fluctuations, and this complicates both validation and interpretation of performance. For event labels, inconsistencies in clinical definitions, sampling schedules, and observer protocols can inflate reported performance in one study while reducing transferability to other farms. Practical reliability, therefore, requires both good reference data and transparent reporting of how labels were produced.

### Reporting standards

To increase both scientific and practical value, performance reporting in PLF biosensing should follow a concise, standardized structure that makes results comparable across studies and interpretable for farm adoption.

First, authors should provide an operational definition of the target phenotype, including the time window, aggregation rule, and the intended decision point, for example, whether the output is meant to trigger additional inspection or to justify immediate treatment. Second, the reference method used to establish the gold standard should be reported transparently, including who performed the labeling, whether assessors were blinded, inter-observer reliability when relevant, and how ambiguous cases were handled, because label quality sets an upper bound on achievable performance. Third, sampling resolution and temporal alignment should be explicitly stated, including the sensor sampling frequency, aggregation interval, and synchronization between sensor streams and reference measurements, since timing mismatches can introduce apparent bias even when the sensor is technically accurate. Fourth, deployment context must be described in sufficient detail to support reproducibility, including housing type such as tie-stall, free-stall, or grazing, barn layout, and management routines, and it should be clarified whether training and evaluation occurred within the same context or across contexts, because housing and routine differences can materially shift signal distributions [33,34]. Fifth, data completeness should be treated as a core performance attribute by quantifying missingness and failure modes attributable to detachment, contamination, occlusion, and communication loss, and by reporting how missingness was handled analytically. Sixth, the validation design should be reported in a way that reveals generalizability, specifying whether the evaluation was within-animal, within-farm, across-farm, or across-region, and whether performance was assessed on unseen farms, seasons, or management systems. Finally, studies should report operational thresholds and practical outputs, including the threshold-selection rule and expected alert burden, such as false positives per 100 cow-days for alerting systems, because practical usability depends on workflow burden and the cost of false alarms, and long-term deployments should include drift monitoring and evidence that performance remains stable over time.

These items align performance reporting with real deployment concerns, such as stable placement, harsh-condition resistance, long-term consistency, and reference data acquisition, all of which have repeatedly been identified as core directions for improving reliability.

## IMPLEMENTATION CONSIDERATIONS AND PRACTICAL LIMITATIONS

Successful PLF biosensing depends on more than nominal model accuracy. Long-term adoption requires that systems remain reliable under harsh barn conditions, fit existing workflows, protect animal welfare, and deliver economic value. Practical limitations often arise at multiple layers, including signal quality, sensor attachment and durability, communication and power, and the usability of alerts and interfaces.

### Data quality and long-term stability

Noise, artifacts, and preprocessing are unavoidable because livestock environments produce motion artifacts, background noise, and intermittent interference. Signal processing elements such as filtering, amplification, and analog-to-digital conversion are essential for improving signal-to-noise ratio and minimizing artifacts, and these choices can meaningfully change downstream model performance [16,89].

Missingness and contamination are common for wearable sensors. For example, leg-mounted devices may be covered by mud, and ear tags can fall out, creating systematic data gaps that degrade both alerting performance and longitudinal trend interpretation [13]. Near-animal systems also face cleanliness and maintenance constraints; automated weighing platforms require frequent manure cleaning to maintain measurement quality [90].

Calibration drift is a central challenge for biochemical and internal sensors. Ruminal pH sensors have been reported to operate for limited periods, for example, around 90 d, due to calibration issues [69]. Recent evidence indicates that electrochemical pH sensors are particularly susceptible to biofouling from protein-rich diets, and that practical pH monitoring capabilities typically deteriorate after approximately 80 to 90 d of deployment, considerably shorter than manufacturer-advertised lifespans [91]. Additionally, proprietary algorithms used by third-party service providers to process raw pH data can create transparency issues, as end users typically receive only interpreted outputs rather than access to raw sensor readings [91,92]. Implantable approaches also face calibration drift and biocompatibility challenges that can limit routine farm deployment, even when data continuity is attractive. These issues imply that reliability reporting should include drift rate, recalibration procedures, and performance decay over time, not only short-term validation.

### Animal welfare and ethical acceptability

Wearable and insertable biosensors can improve welfare by enabling earlier disease detection and faster treatment, but they can also raise welfare concerns about comfort, fit, and invasiveness. Field observations have reported that some cows show mild discomfort when sensors remain in the vagina, even when no clinical disease is observed [57,58]. These considerations support a practical recommendation to evaluate not only detection performance but also retention, skin integrity, behavioral disturbance, and user handling burden when selecting sensor types for routine use.

### Communication and power constraints

Reliability is often limited by communication and power, especially for continuous monitoring and internal devices. Many wearable biosensors rely on batteries, and there is an inherent tradeoff between sampling resolution and battery life. For example, in our previous trial, a noseband sensor operated for only about two weeks before battery replacement was required (unpublished data). Power strategies such as energy harvesting and thermoelectric generation have been explored, and wireless power transfer is under investigation for implanted devices, but these solutions are not yet broadly standardized for livestock deployment [18–20].

Communication dropouts can be a decisive bottleneck. For devices placed deep inside the body, body blocking can cause data transmission failures, and field implementations have explicitly reported communication dropouts due to body blocking signals [73]. This indicates that connectivity planning, network redundancy, and barn-scale wireless design should be considered part of biosensor selection and validation, not an afterthought.

### Standardization and reproducibility across farms

A recurring barrier to scaling PLF biosensing is limited reproducibility across farms and contexts. Differences in housing, management routines, and population characteristics can shift signal distributions and degrade performance when models are transferred without adaptation. This is one reason why sector-level standard-setting bodies are important. For example, the International Committee for Animal Recording is described as setting standards for dairy sensor systems to improve farmers’ confidence in data accuracy. The lack of data standardization and interoperability between sensor systems has been identified as a major obstacle to cross-platform integration, preventing the creation of large-scale comprehensive datasets needed for AI model training [2,91]. Adopting open-source PLF frameworks and standardized data exchange protocols could help overcome this fragmentation.

From an implementation perspective, standardization should include not only device-level accuracy but also consistent phenotype definitions, reporting of data completeness, and clear descriptions of deployment context.

### Alarm fatigue and farmer trust

Even high-performing systems can fail in practice if they create excessive alerts. Information overload and frequent alarms have been identified as key adoption barriers, and repeated false alarms can lead farmers to ignore alerts, resulting in stress and reduced trust in the system [8,93]. This technological burden has been linked to broader concerns among producers about the potential loss of traditional observation skills and growing dependence on PLF tools, underscoring the need for balanced implementation that preserves husbandry expertise while embracing technological advancement [91]. This highlights that alert design must be treated as an implementation problem. Practical approaches include limiting alerts to actionable events, using confidence levels or priority tiers, and reporting expected alert rates under real prevalence, rather than only sensitivity and specificity.

Interfaces and integration also matter. Automated notifications via SMS, email, or farm management software can support timely action, but they can also amplify alert fatigue if not tuned to workflow and decision thresholds [31]. Therefore, deployment should include feedback loops with farmers to refine thresholds, define what constitutes an actionable alert, and ensure that outputs are interpretable and trusted.

### Cost-effectiveness and return on investment

Implementation decisions are ultimately constrained by cost-effectiveness, including capital cost, maintenance labor, energy use, and the value of avoided disease and saved labor. Economic estimates illustrate that automated milking systems can increase productivity and reduce energy consumption, with investment payback depending on labor costs, herd size, and years of operation [94–96]. Feed and live-weight measuring systems have been associated with large reductions in feed waste and feed costs, as well as reduced labor requirements, although increased energy consumption has also been reported [97,98].

These examples support two practical conclusions. First, return on investment depends strongly on the production context and on whether the system reduces labor or prevents costly health events. Second, reviews should encourage studies to report not only technical performance but also operational costs, maintenance needs, and workflow effects, because these factors often determine adoption more than marginal gains in detection accuracy.

### Ethics, data governance, and farmer-animal relationship

Beyond technical reliability and economics, ethical and social acceptability considerations can shape whether biosensor systems are adopted and sustained in commercial dairy farms. Over-reliance on automated sensing and decision support may increase vulnerability to system failures; implementation plans should therefore include fail-safe procedures and clear human oversight in routine management. Frameworks analogous to the General Data Protection Regulation have been proposed for agriculture to ensure that producers retain control over their data while enabling secure, standardized sharing [91]. However, as PLF systems generate increasingly granular individual-level data, questions about who bears the cost and responsibility of proper data storage become additional considerations for adoption.

Biosensor-enabled automation can also change how farmers interact with animals. Reduced direct observation may increases the risk of reduced attentiveness, while simultaneously motivating the development of a new form of human-animal relationship that integrates sensor outputs with attentive husbandry and animal-centered care [93,99]. Practical mitigation strategies include maintaining scheduled visual checks, designing interfaces that promote interpretability rather than black-box recommendations, and involving farmers in threshold selection and alert policies so that technology supports, rather than replaces, skilled stockmanship.

Data governance is increasingly relevant as biosensors generate continuous, individual-level data streams. Key questions include data ownership, consent for secondary use, data security, and who can access or commercialize farm data. Clear contracts, role-based access control, and cybersecurity-by-design are essential to protect sensitive information. At the same time, the field would benefit from mechanisms that enable privacy-preserving data sharing for benchmarking, model validation, and public-good research (e.g., aggregation, anonymization, or federated learning), so that utility can be maximized without undermining farmer trust.

The main implementation issues, mitigation priorities, and reporting items are summarized in Table 3.

## FUTURE DIRECTIONS IN SMART LIVESTOCK FARMING

The next frontier of biosensors in PLF is not simply adding more devices but enabling individualized care through continuous data streams that translate into timely, actionable decisions with minimal unnecessary veterinary intervention. This direction is aligned with precision feeding and targeted medication at the individual-animal level, with the additional promise of reducing antibiotic use while improving welfare. To reach this goal, future advances must integrate robust sensing, reliable connectivity, scalable analytics, and farm-ready decision automation.

### Artificial intelligence and machine learning with edge and cloud computing

The application of AI-driven analytics will increasingly convert raw sensor streams into higher-level biological inference and decision support, particularly for prediction tasks such as disease, estrus, and heat stress risk. The rapid expansion of computer vision in cattle monitoring is a clear signal of this trajectory, and its growth has been linked to advances in machine learning and deep learning for image processing. In cattle specifically, deep-learning algorithms such as convolutional and recurrent neural networks have demonstrated promising accuracy for predicting intake, body weight, body condition score, and disease indicators from RGB-D and thermal images [81], while broader challenges of model transferability, explainability, and participatory design across diverse livestock systems have been critically examined [82]. A practical computing direction is a hybrid edge-and-cloud architecture. Local edge processing can improve resilience when connectivity is unstable and reduce bandwidth for high-frequency streams, while cloud platforms can support long-horizon learning, benchmarking, and integration across systems. In parallel, explainability and transparency will become more important as farms rely on algorithmic recommendations, because trust depends on whether alerts are interpretable and consistent under changing contexts. The concept of hybrid intelligent mechanistic models, which integrate data-driven AI with concept-driven mechanistic frameworks through parallel hybridization, offers a promising pathway for biosensor data analytics that retains biological interpretability while achieving improved predictive accuracy [92,100]. Such hybrid approaches could address the challenge of converting continuous biosensor data streams into biologically meaningful predictions for on-farm decision support.

### Internet-of-Things integration and interoperability

Interoperability is a core bottleneck for scaling PLF because meaningful decisions often require combining signals across devices and platforms. The IoT-integrated biosensors are expected to strengthen real-time centralized herd management by improving data collection, storage, and analysis. In addition, technologies such as blockchain have been proposed to support data integrity and traceability across the supply chain, which may become increasingly important for food safety and quality assurance. However, interoperability also requires sector-level standards. Standard-setting bodies such as International Committee for Animal Recording have been described as important for improving farmer confidence in sensor data quality and comparability across systems. A practical future direction is therefore the convergence of common phenotype definitions, standardized data formats and metadata, and transparent reporting of data completeness and failure modes, so models and performance claims remain reproducible across farms. Edge computing solutions that process data locally before transmitting summarized information when connectivity becomes available have shown promise for enabling sophisticated monitoring even in remote production environments [91], which is particularly relevant for extending dairy PLF beyond intensive indoor systems.

### Digital twins and decision automation

A major conceptual shift is moving from monitoring toward decision automation, where sensor data are transformed into management indices that can trigger or recommend actions. Digital twin approaches that combine real-time biosensor data with mechanistic models to simulate individual animal states before implementing management changes represent an emerging application of this integration [91], and validation of such frameworks will require multi-level assessment of both predictive accuracy and biological plausibility. The proposed data-dimension framework provides a roadmap in which raw signals become first-dimensional sensor data, then second-dimensional quantified measures, then third-dimensional physiological status, and finally a fourth-dimensional decision-support index produced through integration with other information streams. This naturally connects to digital twin concepts, in which each animal has a dynamic state representation updated by multimodal sensor inputs, enabling scenario testing and individualized recommendations. In practice, the near-term frontier is not full autonomy, but robust semi-automation where decision-support outputs are reliable under missing data, drift, and changing environments, and where interventions are constrained by welfare and farm workflow.

### Sustainability and climate-smart farming

Sustainability goals will push biosensing beyond productivity and health into environmental monitoring and mitigation. Ambient sensing of gases such as methane, ammonia, and carbon dioxide has already been positioned within near-animal monitoring, supporting air-quality control and broader sustainability metrics. Climate-smart PLF will likely combine environmental sensing with animal responses (for example, heat stress indicators) to optimize ventilation and cooling in real time. Importantly, sustainability should be evaluated at the system level. For example, biosensor-enabled automation has been discussed alongside estimated reductions in global warming potential under specific adoption scenarios, illustrating the need to quantify environmental outcomes as part of technology assessment [101].

### Genetic selection using longitudinal sensor phenotypes

One of the most transformative opportunities is using biosensors to generate high-resolution, long-term phenotypes that are otherwise too costly or subjective to measure repeatedly. High-resolution data can support prediction of individual needs, including nutrient requirements, and enable individualized care. When combined with genetic insights, biosensor-derived phenotypes can inform selective breeding programs targeting disease resistance, feed efficiency, and other desirable traits, linking PLF monitoring directly to genetic improvement. For this pathway to mature, the field will need stable phenotype definitions, multi-farm reference datasets, and reporting standards that make longitudinal traits comparable across herds and environments.

## CONCLUSIONS

Biosensors in PLF are rapidly expanding from conventional biochemical sensing to multimodal recognition elements that include behavioral, video, and audio-based monitoring. Across at-animal, near-animal, and from-animal approaches, the key scientific and practical challenge is no longer whether signals can be captured, but whether they can be transformed into reliable, interpretable information that supports consistent decisions under farm conditions. Over the next 5 yr, progress will depend on improving robustness and long-term consistency, strengthening reference data acquisition, and integrating biosensors into cloud-based management systems with AI-assisted decision support. Interoperability and standardization will be essential to ensure that models and performance claims transfer across farms and production systems. If these requirements are met, biosensors will enable individualized care through precision feeding and targeted medication, reduce unnecessary interventions, and contribute to welfare and sustainability outcomes. Ultimately, the most durable impact will come not from sensors themselves, but from a decision-connected system that links sensing to analytics, visualization, and action in a trustworthy and economically viable workflow.

## Figures and Tables

**Table 1:** Phenotype-centered map of biosensing in dairy precision livestock farming

Measured phenotype	Sensor modality	Sensing locus	Typical measured outputs	Decision-use point
Activity, lying, standing, walking	Accelerometer wearables	At-animal	Activity index, lying time, step, and movement patterns	Routine surveillance and deviation screening
Eating and rumination	Noseband sensors; accelerometer wearables; acoustic systems	At-animal or near-animal	Eating time, rumination time, and chewing signatures	Precision feeding support and early follow-up
Body weight	Automated weighing systems	Near-animal	Body weight and weight change	Transition monitoring and nutritional management
Body condition score	Computer vision systems	Near-animal	Body condition score and body shape measures	Metabolic risk screening and ration adjustment
Lameness	Computer vision systems; accelerometer wearables	Near-animal or at-animal	Gait, posture features, and locomotion deviation indices	Early warning and prioritization for inspection
Mastitis	Milk-based sensing	From-animal	Screening (EC and SCC) and confirmation (milk biomarkers and pathogen-related assays)	Alerting, diagnostic support, and treatment decision
Ketosis	Milk-based sensing	From-animal	β-hydroxybutyrate	Metabolic disorder screening
Estrus	Accelerometer wearables; computer vision systems; milk-based sensing	At-animal, near-animal, or from-animal	Activity rise, mounting-related behaviors, and progesterone patterns	Optimal timing of insemination
Calving	Vaginal sensors	At-animal	Vaginal temperature patterns and expulsion-based events	Calving supervision and timely assistance
Heat stress	Ambient sensing systems; rumen bolus sensors; computer vision systems	Near-animal or at-animal	THI, internal temperature, and thermal patterns	Cooling and ventilation decision support
Air quality and emissions	Ambient sensing systems	Near-animal	Temperature, humidity, carbon dioxide, ammonia, and methane	Ventilation control and climate-smart monitoring

EC, electrical conductivity; SCC, somatic cell counts; THI, temperature-humidity index.

**Table 2:** Performance reporting for on-farm reliability

Output type	Typical task	Key metrics	Operational reporting	Practical meaning
Event detection	Estrus, mastitis, and ketosis alerts	Se, Sp, PPV, NPV, and prevalence	Threshold, alerts/day, and FP/100 cow-days	Alert workload and trust
Time-critical event detection	Calving and heat stress action	Se, PPV within time window, and lead time	Lead time, miss rate, and false-alert rate	Timing versus false alarms
Continuous prediction	Rumination, eating, and lying	CCC, RMSE or RMSEP, and bias	Sampling, aggregation, and alignment	Threshold usability
Continuous trend monitoring	Walkover weight andvision BCS	CCC, RMSE or RMSEP, and bias	Cleaning rules, outliers, and daily value	Context and preprocessing
Biomarker-based measurement	Milk BHB, progesterone, EC and SCC	Agreement and diagnostic accuracy	Drift, calibration, and sampling protocol	Long-term stability

Se, sensitivity; Sp, specificity; PPV, positive predictive value; NPV, negative predictive value; FP, false positives; cow-days, number of cows multiplied by days observed; CCC, concordance correlation coefficient; RMSE, root mean square error; RMSEP, root mean square error of prediction; BCS, body condition score; BHB, β-hydroxybutyrate; EC, electrical conductivity; SCC, somatic cell count.

**Table 3:** Implementation issues and recommended reporting for farm deployment

Practical issue	Typical failure mode	Mitigation focus	What to report
Data quality	Noise, artifacts, and missingness	Quality control, QA flags, and missingness handling	QC criteria and missingness rate
Sensor retention and contamination	Detachment and fouling by mud or manure	Attachment design and maintenance plan	Retention rate and cleaning frequency
Calibration drift	Long-term signal shift	Drift monitoring and recalibration	Drift rate and recalibration interval
Animal welfare	Discomfort, invasiveness, and handling burden	Welfare screening and low-burden design	Welfare indicators and adverse events
Communication instability	Body-blocking, dead zones, and latency	Gateway planning and buffering	Dropout rate and latency
Power limitation	Battery replacement burden	Duty cycling and wired power	Battery life and maintenance schedule
Standardization and reproducibility	Phenotype definition mismatch and farm context shift	External validation and context reporting	Housing description andvalidation design
Alarm fatigue	Excess false alerts	Threshold tuning and alert tiering	Alerts/day and FP/100 cow-days
Cost-effectiveness	Unclear ROI	Benefit tracking	Cost items, labor saved, and outcomes

QA, quality assurance; QC, quality control; FP, false positives; cow-days, number of cows multiplied by days observed; ROI, return on investment.

## Data Availability

Upon reasonable request, the datasets of this study can be available from the corresponding author.
